# Oral antibiotic use and early-onset colorectal cancer: findings from a case-control study using a national clinical database

**DOI:** 10.1038/s41416-021-01665-7

**Published:** 2021-12-17

**Authors:** Ronald McDowell, Sarah Perrott, Peter Murchie, Christopher Cardwell, Carmel Hughes, Leslie Samuel

**Affiliations:** 1 Centre for Public Health, School of Medicine, Dentistry & Biomedical Science, Queen’s University, Belfast, Northern Ireland; 2 School of Medicine, Medical Sciences and Nutrition, University of Aberdeen, Aberdeen, Scotland; 3 School of Pharmacy, Queen’s University, Belfast, Scotland; 4 Department of Clinical Oncology, Aberdeen Royal Infirmary, NHS Grampian, Aberdeen, Scotland

**Keywords:** Cancer epidemiology, Colon cancer, Colon cancer, Cancer epidemiology

## Abstract

**Background:**

Antibiotic-induced gut dysbiosis has been associated with colorectal cancer (CRC) in older adults. This study will investigate whether an association exists between antibiotic usage and early-onset colorectal cancer (CRC), and also evaluate this in later-onset CRC for comparison.

**Methods:**

A case-control study was conducted using primary care data from 1999–2011. Analysis were conducted separately in early-onset CRC cases (diagnosed < 50 years) and later-onset cases (diagnosed ≥ 50 years). Conditional logistic regression was used to calculate odds ratios and 95% confidence intervals (CI) for the associations between antibiotic exposure and CRC by tumour location, adjusting for comorbidities.

**Results:**

Seven thousands nine hundred and three CRC cases (445 aged <50 years) and 30,418 controls were identified. Antibiotic consumption was associated with colon cancer in both age-groups, particularly in the early-onset CRC cohort (<50 years: adjusted Odds Ratio (OR_adj_) 1.49 (95% CI 1.07, 2.07), *p* = 0·018; ≥50 years (OR_adj_ (95% CI) 1.09 (1.01, 1.18), *p* = 0·029). Antibiotics were not associated with rectal cancer (<50 years: OR_adj_ (95% CI) 1.17 (0.75, 1.84), *p* = 0.493; ≥50 years: OR_adj_ (95% CI) 1.07 (0.96, 1.19), *p* = 0.238).

**Conclusion:**

Our findings suggest antibiotics may have a role in colon tumour formation across all age-groups.

## Background

Since the late 1980s, global antibiotic consumption and cases of early-onset colorectal cancer (CRC) have increased markedly [1–3]. This pattern may be related; antibiotic consumption has been associated with CRC genesis in adults of all ages [4–8]. In contrast to declining incidence amongst older populations [5, 9], CRC incidence among adults aged 20–29 years in Europe is increasing by ~8% each year [1]. In the USA, CRC is the second most common incident cancer and third leading cause of cancer death in adult males less than 50 years old [10]. Consensus exists that early-onset CRC (<50 years) is different to later-onset CRC (≥50 years) in terms of epidemiology, pathology and biology [5, 6, 11], although more recent evidence suggests both types are clinically and genomically indishtinguishable [12]. Therefore, there may be a rationale for studying early-onset CRC separately from later-onset CRC to identify specific risk factors associated with the rising trend observed among younger people.

Worldwide, there were ~70 billion doses of antibiotics consumed in 2011—which equates to 10 per person on earth [13]. Although essential for many medical interventions, children and teenagers are amongst those most commonly exposed to antibiotic therapy [14] and may be more vulnerable to the potential effects of overexposure—such as obesity, allergic diseases and inflammatory bowel disease [5, 15]. In the USA, 69% of children aged less than 2 years are exposed to antibiotics [16], and as accessibility to antibiotics increases across low and middle-income countries, antibiotic usage for common childhood infections is becoming more widespread [17, 18]. Furthermore, high prevalence of acne amongst adolescents can result in long-term antibiotic exposure, sometimes lasting months to years due to varying national guidelines and uncertainty regarding optimum treatment duration [19]. In addition, at least 20–30% of antibiotics prescribed in primary care may be inappropriate [18, 20].

The relationship between pathogenic organisms and cancer is well-established; *Helicobacter pylori* is associated with gastric cancer and human papilloma virus (HPV) with anal, cervical, tonsillar and vulval cancer [21, 22]. Antibiotic-induced microbiome changes can be permanent and irregularities in immunostimulatory bacterial products can impede normal immune-surveillance, increasing the risk of carcinogenesis [21]. In addition, interruption of normal gut commensals may allow colonisation by pathogenic bacteria, which invade and damage the gut mucosa, leading to inflammation and tumour formation [22]. Examples of these harmful microbes include strains of *Escherichia. coli* and *Bacteroides. fragilis*; which may be promoted by certain antibiotics [22, 23].

Several single-centred studies [4, 24–32] and two recent systematic reviews and meta-analyses [7, 8] found an association between oral antibiotics CRC risk. However, these studies are contradictory; whether risk varies with tumour location or with use of anti-aerobic or anti-anaerobic agents (i.e. antibiotics active against bacteria requiring oxygen, or lack of oxygen, to survive) or both, remains uncertain. To our knowledge, no study before has investigated antibiotic usage in the early-onset CRC population; participant mean age is ~69–72 years in all recorded studies [4, 24–32].

This study seeks to determine the association between antibiotic use and early-onset CRC, and whether any risk may differ within the colorectal continuum, or by antibiotic spectrum of activity.

## Materials and methods

### Data source

Study data were obtained from the population-based Primary Care Clinical Information Unit Research (PCCIUR) database [33], comprising over two million patients registered at 393 general practices across Scotland between 1993 and 2011. PCCIUR contains up to 20 years of demographic, clinical and diagnostic information and has been widely used in epidemiological research [34–37].

### Study design

A case-control study was conducted using PCCIUR data. Cases were patients with a new diagnosis of primary CRC (Read codes B13, B14, see Supplementary Material Table S1) between 1999 and 2011. Cases were excluded if they had a previous cancer, excluding non-melanoma skin cancer, or were diagnosed with other primary cancers on the date of diagnosis due to uncertainty about the primary cancer and the potential for coding errors. Cases of anal cancer were excluded as they are squamous cell cancers and associated with HPV infection. Patients with diagnosed conditions predisposing to CRC (e.g. inflammatory bowel disease, Peutz-Jeghers syndrome, polyposis syndromes) were excluded as our study was limited to sporadic CRC. Patients with diagnosed immunosuppressive states (e.g. Sjogren’s syndrome, HIV infection, transplantation) and those in receipt of immunosuppressive medicines during the exposure period (see definition below) were also excluded.

**Fig. 1:**
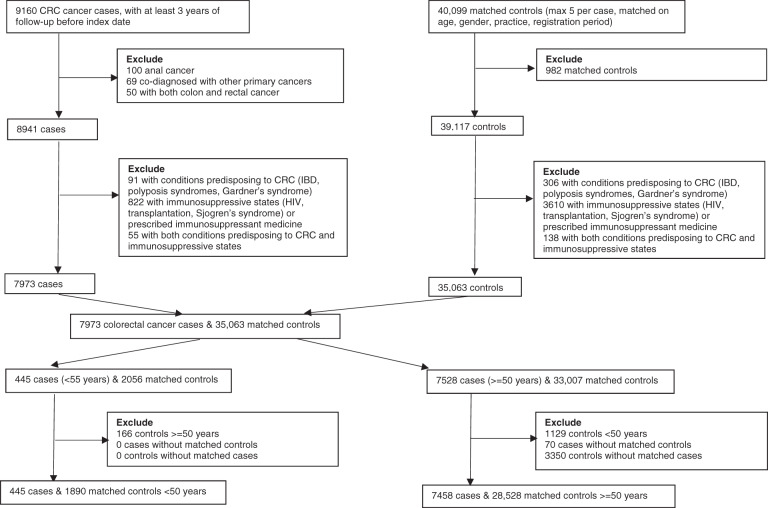
Data extraction flow chart. CRC: colorectal cancer; IBD: inflammatory bowel disease.

All available controls (alive, registered with a GP and free from cancer (excepting non-melanoma skin cancer)) were identified for each case matching on practice, year of birth (±5 years), gender and year of registration (in categories). Up to five controls for each case were randomly selected from those available, without replacement. The index date within each matched set was defined as the diagnosis date of CRC in the case. Both cases and controls needed at least three years of follow-up data and remained registered with the same general practice over the follow-up period. Two strata were constructed for comparative purposes, a younger strata (cases plus matched controls <50 years) and an older strata (cases plus matched controls ≥ 50 years) [5, 6, 38]. Data extraction are depicted in Fig. 1.

Within each matched set, the exposure period, i.e. the period of time over which medicine use was determined, started on either 1 January 1993 (as prescriptions before this time were unlikely to be recorded electronically), or the most recent GP registration date if this occurred after January 1993. This ensured all members within each matched set had the same exposure period. The exposure period ended 1 year before the index date, to reduce the risk of reverse causality and exclude medications unlikely to have had sufficient time to cause cancer [39]. Rectal or rectosigmoid junction tumours were classified as rectal cancer, otherwise tumours were classified as colon cancer.

### Classification and definition of antibiotic exposure

Prescriptions for oral antibiotics were extracted from PCCIUR. These were classified by drug class and by presence or absence of anti-anaerobic effects to provide insight into bacterial populations potentially associated with CRC [4]. Medicines studied are listed in the Supplementary Material Table S2.

For each antibiotic prescription, the duration of treatment (in days) was identified from prescribing records. Where this was not recorded (*n* = 536 (1.1%) of all antibiotic prescriptions), treatment duration was estimated according to standard dosing for each antibiotic. Total exposure in days of all antibiotic classes was calculated for each patient and categorised as 0 days, 1–15 days, 16–60 days, >60 days [40, 41]. Analyses were also conducted using cumulative duration of anti-anaerobic or non-anti-anaerobic antibiotic treatment. For these analyses only primary clinical therapeutic effect(s) of each medicine were considered; other antimicrobial activity is often less pronounced without major effects on aerobic or anaerobic populations [42].

### Covariates

The following comorbidities, based upon published Read codes for the Charlson Comorbidity Index (CCI) [43], were identified prior to or during the exposure period: diabetes, myocardial infarction, coronary heart disease, heart failure, peripheral vascular disease, dementia, cerebrovascular disease, chronic obstructive pulmonary disease, osteoporosis, renal disease, liver disease and hemiplegia/paraplegia. Additional comorbidities, relevant to CRC (i.e. gallstones, acromegaly), were also identified. We also adjusted for use of low dose aspirin and non-steroidal anti-inflammatory drugs (NSAIDs), as these may reduce risk of CRC [44, 45]. Smoking status (non-smoker, current smoker, former smoker) [46] and alcohol consumption (non-drinker, light or moderate drinker, heavy drinker) [47] were determined from the most recent smoking or alcohol record prior to or during the exposure period.

### Statistical analysis

Descriptive statistics summarised cases and controls. For each cohort, conditional logistic regression was used to calculate odds ratios (OR) and 95% confidence intervals (CI) for associations between each exposure and CRC, with adjustment for comorbidities. The matched design accounted for age (±5 years), GP practice, gender and year of registration. All analyses were adjusted for age in years, as participants were matched in age bands rather than by calendar year. Interaction tests to determined whether antibiotic exposure effects varied by strata. To test for trend in risk of colorectal cancer across different categories of treatment length, the duration of antibiotic exposure was treated as a continuous rather than a categorical variable. Associations between individual classes of antibiotics and colon/rectal cancer are reported as supplementary analyses due to low prescribing levels of individual classes among patients under 50 years and the increased risk of type 1 errors due to multiple testing.

### Subgroup analyses

Analyses were repeated for matched sets where location of cases’ colon tumour was explicitly recorded in the diagnostic readcodes, namely proximal colon (malignant neoplasms of hepatic flexure, transverse colon, caecum, appendix or ascending colon) and distal colon (malignant neoplasms of the descending colon, sigmoid colon or splenic flexure of colon). The primary analyses were repeated using the subsample of patients with recorded body mass index (BMI).

### Sensitivity analyses

Sensitivity analyses were undertaken as follows: (1) period of time before index date during which prescriptions were not counted was increased from 1 to 2 years to reduce potential for reverse causation; (2) threshold used to distinguish between younger and older patients was lowered from 50 years to 45 years; (3) threshold used to distinguish between younger and older patients was increased from 50 years to 55 years; (4) adjustments were made for comorbidities, smoking and alcohol use for the 23,702 patients (61.2%) where both lifestyle factors had been recorded in the patient’s clinical records. The latter analysis was also repeated using multiple imputation with chained equations (MICE) techniques to impute smoking and alcohol status. This is a simulation-based method appropriate for handling missing data assuming that such values are missing at random. Ordered logit models were used with age, gender, deprivation within the GP practice locality, and comorbidities for the imputations, stratified by case-control status and using 25 imputations [48].

## Results

### Descriptive statistics: cases and controls

Seven thousands nine hundred and three CRC cancer cases and 30,418 matched controls were identified. Five thousands three hundred fifty six cases (67.8%) had at least four matched controls. There were 5281 colon cancer cases and 2662 rectal cancer cases. Median (inter-quartile range (IQR)) age at diagnosis in the younger and older strata was 45 [41, 47] years and 71 years (63, 78), respectively. The exposure period, matched in cases and controls, was slightly shorter for patients <50 years (median (IQR) 6.9 (4.8, 9.2) years) than patients ≥ 50 years (median (IQR) 7.9 (5.3, 10.8) years). Approximately 55% of patients were male in each age-group.

Characteristics of cases and controls are listed in Table 1. A full set of descriptive statistics of cases and controls by each tumour location is provided as Supplementary Material Table S3.

**Table 1:** Characteristics of cases and controls.

Variable	Category	Cohort			
		<50 years		≥50 years	
		Cases *n* (%)	Controls *n* (%)	Cases *n* (%)	Controls *n* (%)
Number of patients		445	1890	7458	28,528
Length of exposure period (years): median (IQR)		6.9 (4.8, 9.3)	6.9 (4.8, 9.2)	8.0 (5.4, 10.9)	7.8 (5.3, 10.8)
Year of diagnosis/index date: median (IQR)		2004 (2002, 2007)	2004 (2002, 2007)	2004 (2002, 2007)	2004 (2002, 2007)
Age at diagnosis/index date: median (IQR)		45 (41, 47)	42 (39, 45)	71 (63, 78)	69 (61, 77)
Deprivation quintile	1 (least deprived)	48 (10.8%)	207 (11.0%)	888 (11.9%)	3466 (12.1%)
	2	125 (28.1%)	525 (27.8%)	2077 (27.8%)	8005 (28.1%)
	3	38 (8.5%)	159 (8.4%)	699 (9.4%)	2718 (9.5%)
	4	107 (24.0%)	453 (24.0%)	1901 (25.5%)	7192 (25.2%)
	5 (most deprived)	126 (28.3%)	544 (28.8%)	1886 (25.3%)	7122 (25.0%)
	Missing	≤5 (≤1.1%)	≤5 (≤0.3%)	7 (0.1%)	25 (0.1%)
Gender	Male	230 (51.7%)	997 (52.8%)	4153 (55.7%)	15,998 (56.1%)
	Female	215 (48.3%)	893 (47.3%)	3305 (44.3%)	12,530 (43.9%)
Smoking status^a^	Never smoked	165 (37.1%)	660 (34.9%)	2776 (37.2%)	10,235 (35.9%)
	Ex-smoker	48 (10.8%)	138 (7.3%)	1727 (23.2%)	5376 (18.8%)
	Current smoker	102 (22.9%)	446 (23.6%)	1193 (16.0%)	5375 (18.8%)
	Missing	130 (29.2%)	646 (34.2%)	1762 (23.6%)	7542 (26.4%)
Alcohol consumption^a^	Non-drinker	36 (8.1%)	165 (8.7%)	1099 (14.7%)	4056 (14.2%)
	light/moderate	238 (53·5%)	846 (44·8%)	3,563 (47·8%)	13,053 (45·8%)
	heavy drinker	12 (2·9%)	56 (3·0%)	331 (4·4%)	1,086 (3·8%)
	missing	158 (35·5%)	823 (43·5%)	2,465 (33·1%)	10,333 (36·2%)
Comorbidities diagnosed prior to or during the exposure period					
Diabetes		7 (1.6%)	38 (2.0%)	846 (11.3%)	2334 (8.2%)
Myocardial infarction		0 (0.0%)	10 (0.5%)	544 (7.3%)	1914 (6.7%)
Coronary heart disease		≤5 (≤1.1%)	16 (0.9%)	1337 (17.9%)	4588 (16.1%)
Heart failure		≤5 (≤1.1%)	≤5 (≤0.3%)	299 (5.0%)	954 (3.3%)
Peripheral vascular disease		≤5 (≤1.1%)	9 (0.5%)	355 (4.8%)	1254 (4.4%)
Dementia		0 (0.0%)	0 (0.0%)	57 (0.8%)	444 (1.6%)
Cerebrovascular disease		≤5 (≤1·1%)	9 (0.5%)	593 (8.0%)	2112 (7.4%)
Chronic obstructive pulmonary disease		7 (1.6%)	31 (1.6%)	498 (6.7%)	1712 (6.0%)
Osteoporosis		0 (0.0%)	0 (0.0%)	178 (2.4%)	750 (2.6%)
Renal disease		≤5 (≤1.1%)	≤5 (≤0.3%)	311 (4.2%)	902 (3.2%)
Liver disease		0 (0.0%)	≤5 (≤0.3%)	52 (0.7%)	199 (0.7%)
Hemiplegia/paraplegia		≤5 (≤1.1%)	≤5 (≤0.3%)	46 (0.6%)	162 (0.6%)
Gallstones		8 (1.8%)	23 (1.2%)	483 (6.5%)	1525 (5.4%)
Acromegaly		≤5 (≤1.1%)	0 (0.0%)	0 (0.0%)	≤5 (≤0.1%)
Medication use during exposure period					
Low dose aspirin		11 (2.5%)	33 (1.8%)	2157 (28.9%)	7349 (25.8%)
NSAIDs		128 (28.8%)	483 (25.5%)	2754 (36.9%)	10,646 (37.3%)

*IQR* inter-quartile range, *NSAIDs* non-steroidal anti-inflammatory drugs.

^a^Most recent record in patient’s clinical history prior to 1-year lag.

### Descriptive statistics: antibiotic medication

44.9% (17,206) of patients were prescribed antibiotics during the exposure period. The proportion of CRC cases prescribed antibiotics was larger than the proportion of controls prescribed antibiotics in both the <50 strata (cases: 47.2% (210) v controls: 40.1% (757)) and the ≥ 50 years strata (cases: 46.8% (3496) v controls: 44.7% (12,743)). Most commonly prescribed antibiotics were penicillins (52.8% (25,473) of all antibiotic prescriptions). The proportion of cases prescribed each class of antibiotic was usually higher than the proportion of controls in both age-groups for both colon and rectal cancer.

Antibiotics with anti-anaerobic effects were more commonly prescribed than antibiotics without anti-anaerobic effect (52.7% (25,440) v 47.3% (22,851)). Prescribing of both anti-anaerobic antibiotics and non-anti-anaerobic antibiotics was higher among cancer cases than controls in both age-groups and cancer sites. Descriptive statistics for class of antibiotic medication by age-group and tumour location are given in Table 2.

**Table 2:** Antibiotic use, by class, in cases and controls.

		<50 years		≥50 years	
Site	Medicine	Cases *n* (%)	Controls *n* (%)	Cases *n* (%)	Controls *n* (%)
Colon	Cephalosporins	19 (6.6%)	64 (5.3%)	417 (8.4%)	1463 (7.7%)
	Macrolides	33 (11.4%)	125 (10.3%)	575 (11.5%)	2030 (10.6%)
	Penicillins	105 (36.3%)	369 (30.3%)	1884 (37.7%)	6742 (35.4%)
	Quinolones and nalidixic acid	14 (4.8%)	38 (3.1%)	258 (5.2%)	1044 (5.5%)
	Sulpha and trimethoprim	26 (9.0%)	87 (7.2%)	539 (10.8%)	1937 (10.2%)
	Tetracyclines	27 (9.3%)	103 (8.5%)	360 (7.2%)	1362 (7.1%)
	Other	13 (4.5%)	48 (3.9%)	192 (3.9%)	704 (3.7%)
Rectum	Cephalosporins	9 (5.8%)	27 (4.0%)	158 (6.4%)	622 (6.6%)
	Macrolides	19 (12.2%)	71 (10.6%)	234 (9.5%)	862 (9.1%)
	Penicillins	55 (35.3%)	214 (31.8%)	819 (33.2%)	3058 (32.3%)
	Quinolones and nalidixic acid	6 (3.9%)	20 (3.0%)	116 (4.7%)	432 (4.6%)
	Aulpha & trimethoprim	12 (7.7%)	36 (5.4%)	216 (8.8%)	781 (8.3%)
	Tetracyclines	12 (7.7%)	60 (8.9%)	150 (6.1%)	638 (6.8%)
	Other	8 (5.1%)	29 (4.3%)	79 (3.·2%)	332 (3.5%)

### Associations between use of antibiotics and CRC

Use of antibiotics was associated with increased risk of colon cancer in both age-groups (<50 years: adjusted Odds Ratio (OR_adj_) 1.49 (95% CI 1.07, 2.07), *p* = 0·018; ≥50 years: OR_adj_ 1.09 (95% CI 1.01, 1.18), *p* = 0.029)) (Table 3). Although this effect was greater among patients <50 years compared to those ≥ 50 years, the difference was not significant (interaction test: *p* = 0.071). There was no apparent exposure-response relationship between antibiotic use and colon cancer risk for either age-groups (<50 years: *P-*trend *p* = 0.177; ≥50 years: *P-*trend *p* = 0.082). Antibiotics use was not significantly associated with increased rectal cancer risk in either age-group (<50 years: OR_adj_ 1.17 (95% CI 0.75, 1.84), *p* = 0.493; ≥50 years: OR_adj_ 1.07 (95% CI 0.96, 1.19), *p* = 0·238)).

**Table 3:** Associations between antibiotic use and colorectal cancer.

Primary analyses										
		<50 years				≥50 years				
Site	Medicine	Cases *n* (%)	Controls *n* (%)	Adjusted^b^ OR (95% CI)	Adjusted^b^ *p*-value	Cases *n* (%)	Controls *n* (%)	Adjusted^b^ OR (95% CI)	Adjusted^b^ *p*-value	Interaction test *p*-value
Colon	Any antibiotic use	141 (48.8%)	492 (40.4%)	1.49 (1.07, 2.07)	0.018	2422 (48.5%)	8775 (46.0%)	1.09 (1.01, 1.18)	0.029	0.071
	Anti-anaerobic activity^a^	107 (37.0%)	386 (31.7%)	1.34 (0.97, 1.86)	0.078	1827 (36.6%)	6594 (34.6%)	1.08 (1.00, 1.17)	0.054	0.204
	Non-anti-anaerobic activity^a^	91 (31.5%)	303 (24.9%)	1.37 (0.97, 1.93)	0.075	1585 (31.8%)	5741 (30.1%)	1.03 (0.95, 1.11)	0.500	0.151
Rectum	Any antibiotic use	69 (44.2%)	69 (44.2%)	1.17 (0.75, 1.84)	0.493	1074 (43.6%)	3968 (42.0%)	1.07 (0.96, 1.19)	0.238	0.698
	Anti-anaerobic activity^a^	56 (35.9%)	56 (35.9%)	1.11 (0.70, 1.76)	0.646	803 (32.6%)	3026 (32.0%)	1.00 (0.89,1.11)	0.974	0.650
	Non-anti-anaerobic activity^a^	44 (28.2%)	44 (28.2%)	1.70 (1.06, 2.74)	0.029	684 (27.7%)	2499 (26.4%)	1.05 (0.93, 1.18)	0.436	0.888

Subgroup analyses										
Proximal colon	Any antibiotic use	31 (62.0%)	87 (41.0%)	3.78 (1.60, 8.92)	0.002	272 (42.7%)	1048 (43.3%)	0.92 (0.74, 1.13)	0.315	0.001
	Anti-anaerobic activity^a^	22 (44.0%)	64 (30.2%)	2.17 (1.00, 4.68)	0.049	208 (32.7%)	744 (30.8%)	1.10 (0.88, 1.37)	0.537	0.034
	Non-anti-anaerobic activity^a^	19 (38.0%)	53 (25.0%)	2.86 (1.21, 6.73)	0.016	179 (28.1%)	741 (30.7%)	0.79 (0.64, 0.99)	0.036	0.153
Distal colon	Any antibiotic use	20 (55.6%)	60 (40.0%)	3.39 (1.02, 11.28)	0.047	245 (47.6%)	896 (44.2%)	1.14 (0.90, 1.44)	0.289	0.083
	Anti-anaerobic activity^a^	12 (33.3%)	51 (34.0%)	1.06 (0.35, 3.23)	0.922	178 (34.6%)	675 (33.3%)	1.02 (0.80, 1.31)	0.971	0.930
	Non-anti-anaerobic activity^a^	16 (44.4%)	31 (20.7%)	5.17 (1.71, 15.60)	0.004	152 (29.5%)	578 (28.5%)	1.01 (0.79, 1.29)	0.990	0.916

*OR* odds ratio, *CI* confidence interval.

^a^Primary clinical therapeutic effect on gut microbe.

^b^Adjusted for diabetes, myocardial infarction, coronary heart disease, heart failure, peripheral vascular disease, dementia, cerebrovascular disease, chronic obstructive pulmonary disease, osteoporosis, renal disease, liver disease, hemiplegia/paraplegia, gallstones, acromegaly, low dose aspirin and NSAIDs.

**Fig. 2:**
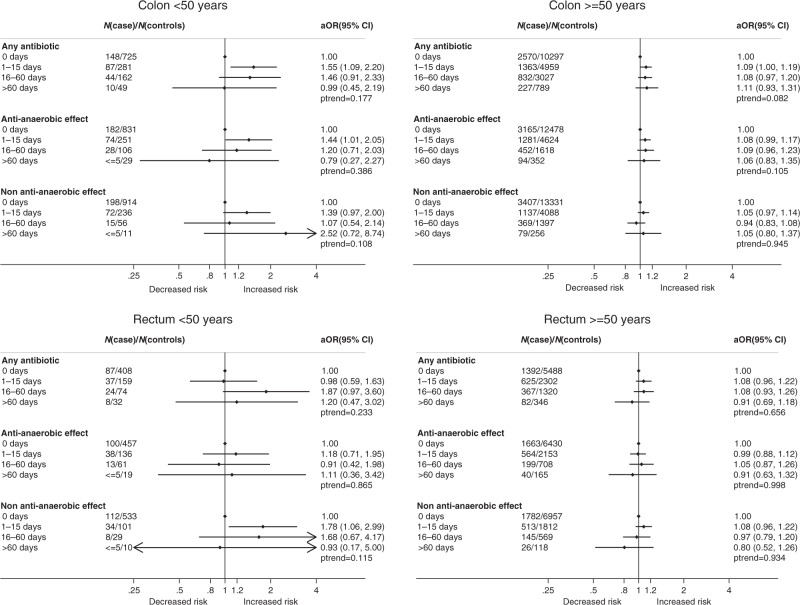
Forest plots for associations between antibiotic use and risk of colorectal cancer. aOR: adjusted Odds Ratio; N: Number analyses adjusted for diabetes, myocardial infarction, coronary heart disease, heart failure, peripheral vascular disease, dementia, cerebrovascular disease, chronic obstructive pulmonary disease, osteoporosis, renal disease, liver disease, hemiplegia/paraplegia, gallstones, acromegaly, low dose aspirin and NSAIDs

Analyses of antibiotic prescribing by treatment duration and CRC location are depicted graphically in Forest Plots (Fig. 2) and listed in Supplementary Material Table S4.

There was no evidence at the 5% statistical significance level of any differences between the the two age-groups in associations between classes of antibiotic use and the risk of colon or rectal cancer (Supplementary Material Table S5).

### Associations between anti-anaerobic effects and CRC

Although not significant, use of anti-anaerobic antibiotics was associated with increased risk of colon cancer in both strata (<50 years: OR_adj_ 1.34 (95% CI 0.97, 1.86), *p* = 0.078; ≥50 years: OR_adj_ 1.08 (95% CI 1.00, 1.17), *p* = 0.054) (Table 3). However, these effects did not differ significantly from each other (interaction test: *p* = 0·204) and there was no apparent exposure-response relationship in either age-group (<50 years: *P-*trend *p* = 0.386; ≥50 years: *P-*trend *p* = 0.105). Antibiotic use was not significantly associated with increased risk of rectal cancer in either age-group (<50 years: OR_adj_ 1.11 (95% CI 0.70, 1.76), *p* = 0.646; ≥50 years: OR_adj_ 1.00 (95% CI 0.89, 1.11), *p* = 0.974)).

### Associations between non-anti-anaerobic effects and CRC

There was some evidence that use of antibiotics without anti-anaerobic effects was associated with increased risk among patients <50 years of both colon cancer (OR_adj_ 1.37 (95% CI 0.97, 1.93), *p* = 0·075) and rectal cancer (OR_adj_ 1.·70 (95% CI 1.06, 2.74), *p* = 0.029), but not among patients ≥ 50 years (colon cancer: OR_adj_ 1.03 (95% CI 0.95, 1.11), *p* = 0.500; rectal cancer: OR_adj_ 1.·05 (95% CI 0.93, 1.18), *p* = 0·436) (Table 3). However, these effects neither vary between age-groups (interaction test: colon cancer *p* = 0.151, rectal cancer *p* = 0.888), nor was there an exposure-response relationship between use of antibiotics without anti-anaerobic effects and either colon or rectal cancer risk in either age-groups.

### Subgroup analyses

There were 687 (13.0%) colon cancer cases classified as proximal colon and 551 (10.4%) classified as distal colon. Use of antibiotics, antibiotics with anti-anaerobic effects and antibiotics without anti-anaerobic effects was associated with increased risk of proximal colon cancer among <50 s (any antibiotic OR_adj_ 3.78 (95% CI 1.60, 8.92), *p* = 0.002; anti-anaerobic OR_adj_ 2.17 (95% CI 1.00, 4.68), *p* = 0.049; non-anti-anaerobic OR_adj_ 2.86 (95% CI 1.21, 6.73), *p* = 0·016). It appeared effects associated with antibiotic use and anti-anaerobic antibiotics differed between the two age-groups (interaction test: any antibiotic *p* = 0.001; anti-anaerobic *p* = 0.034). A positive exposure-response relationship was also observed between antibiotic prescribing and risk of proximal colon cancer among the younger patients (*P-*trend = 0.004)). Results for both subgroup analyses are listed in Table 3 and Supplementary Material Table S6.

One-third of all patients included in our analyses (*n* = 12,657) (33·0%) had their BMI reported, and these patients were on average slightly overweight (median (IQR) 26.7 (23.9,29.9)). Patients with recorded BMI were less likely to be non-smokers or non-drinkers, and have higher reported levels of comorbidities and prescribed medication, than patients where BMI was missing (Supplementary Material Table S7). Adjusting for BMI in addition to comorbidities and medicine use increased the magnitude of the association between any antibiotic use and early-onset colon cancer risk (OR_adj_ 1.98 (95% CI 0.82, 4.81), *p* = 0.130), although this association did not differ significantly between that reported with the older age-group (OR_adj_ 1.01 (95% CI 0.87, 1.16), *p* = 0.920) (interaction test: *p* = 0.139). Full details of these subgroup analyses are reported in Supplementary Material Tables S8 and S9.

### Sensitivity analyses

Results from sensitivity analyses are listed in Table 4. Increasing lag-time from one year to 2 years or additionally adjusting for alcohol and smoking had no substantive impact on reported associations between antibiotic use and CRC risk.

**Table 4:** Sensitivity analyses for associations between antibiotic use and colorectal cancer.

		2-year lag		Adjusted for comorbidities, smoking and alcohol (whole cases)		Adjusted for comorbidities, smoking and alcohol (MI)		Redefine age strata		Redefine age strata	
		<50 years	≥50 years	<50 years	≥50 years	<50 years	<50 years	<45 years	≥45 years	<55 years	≥55 years
Site	Cumulative exposure	Adjusted^a^ OR (95% CI)	Adjusted^a^ OR (95% CI)	Adjusted^a^ OR (95%CI)	Adjusted^a^ OR (95% CI)	Adjusted^a^ OR (95% CI)	Adjusted^a^ OR (95% CI)	Adjusted^a^ OR (95% CI)	Adjusted^a^ OR (95%CI)	Adjusted^a^ OR (95% CI)	Adjusted^a^ OR (95% CI)
Colon	0 days	1.00	1.00	1.00	1.00	1.00	1.00	1.00	1.00	1.00	1.00
	≥1 day	1.42 (1.01, 1.99)	1.04 (0.96, 1.13)	1.46 (0.92, 2.34)	1.00 (0.90, 1.11)	1.50 (1.07, 2.09)	1.08 (1.00, 1.17)	1.79 (1.13, 2.84)	1.10 (1.02, 1.18)	1.22 (0.94, 1.58)	1.10 (1.01, 1.19)
	1–15 days	1.52 (1.05,2.19)	1.05 (0.96, 1.14)	1.52 (0.93, 2.49)	1.00 (0.89, 1.12)	1.56 (1.09, 2.23)	1.08 (1.00, 1.18)	1.85 (1.14, 3.01)	1.10 (1.01, 1.20)	1.21 (0.91, 1.61)	1.11 (1.01, 1.21)
	16–60 days	1.23 (0.74, 2.03)	1.03 (0.92, 1.15)	1.46 (0.76, 2.79)	1.00 (0.87, 1.14)	1.45 (0.90, 2.34)	1.07 (0.96, 1.19)	1.72 (0.90, 3.29)	1.08 (0.98, 1.20)	1.22 (0.85, 1.77)	1.09 (0.98, 1.22)
	>60 days	1.13 (0.49, 2.59)	1.06 (0.88, 1.28)	0.78 (0.23, 2.57)	0.99 (0.80, 1.22)	1.01 (0.46, 2.24)	1.10 (0.93, 1.31)	1.35 (0.46, 4.02)	1.11 (0.94, 1.31)	1.25 (0.70, 2.22)	1.07 (0.90, 1.28)
	*P-*trend	0.285	0.429	0.447	0.949	0.176	0.115	0.098	0.064	0.201	0.980
Rectum	0 days	1.00	1.00	1.00	1.00	1.00	1.00	1.00	1.00	1.00	1.00
	≥1 day	1.08 (0.68, 1.71)	1.06 (0.95, 1.19)	0.87 (0.44, 1.69)	0.99 (0.85, 1.14)	1.16 (0.74, 1.83)	1.06 (0.95, 1.18)	0.94 (0.49, 1.79)	1.07 (0.96, 1.20)	1.29 (0.91, 1.83)	1.06 (0.95, 1.19)
	1–15 days	0.94 (0.57, 1.56)	1.08 (0.95, 1.22)	0.71 (0.34, 1.50)	1.00 (0.85, 1.18)	0.97 (0.58, 1.62)	1.07 (0.95, 1.21)	0.64 (0.29, 1.41)	1.09 (0.97, 1.23)	1.36 (0.94, 1.97)	1·05 (0.93, 1.19)
	16–60 days	1.38 (0.66, 2.91)	1.09 (0.92, 1.28)	1.56 (0.56, 4.38)	1.00 (0.83, 1.22)	1.89 (0.98, 3.65)	1.08 (0.93, 1.26)	1.97 (0.78, 4.95)	1.09 (0.94, 1.27)	1.21 (0.71, 2.06)	1.12 (0.96, 1.31)
	>60 days	1.17 (0.40, 3.40)	0.91 (0.68, 1.22)	1.06 (0.28, 3.96)	0.79 (0.56, 1.12)	1.15 (0.45, 2.92)	0.89 (0.68, 1.16)	0.99 (0.25, 3.89)	0.89 (0.68, 1.16)	0.97 (0.45, 2.09)	0.92 (0.70, 1.22)
	*P-*trend	0.593	0.577	0.830	.490	0.257	0.772	0.588	0.636	0.554	0.479

*OR* odds ratio, *CI* confidence interval, *MI* multiple imputation.

^a^Adjusted for diabetes, myocardial infarction, coronary heart disease, heart failure, peripheral vascular disease, dementia, cerebrovascular disease, chronic obstructive pulmonary disease, osteoporosis, renal disease, liver disease, hemiplegia/paraplegia, gallstones, acromegaly, low dose aspirin and NSAIDs.

## Discussion

In this large population-based case-control study of early-onset CRC cases and later-onset CRC cases, antibiotic consumption was associated with colon cancer pathogenesis across all age-groups.

Results from a systematic review and meta-analysis of 10 high-quality observational studies found antibiotic use increased CRC risk (effect size (ES) 1.17 (95% CI 1.05, 1.30)), but associations differed with tumour location and antibiotic classes [7]. Analysis of colon cancer cases alone showed no significant association (ES 1.06 (95% CI 0.89, 1.26)). However, there was high heterogeneity between studies (I^2^ = 95.7% and 83.5%, respectively), which—if our findings are true—may partly reflect varying and older age-groups included in those studies. Other than colon and rectal cancer, the meta-analysis did not explore the influence of antibiotics on tumour locations further—such as association with proximal colon cancer. Another systematic review and meta-analysis suggested a weak association may exist between antibiotic consumption and risk of CRC [8]. However, definitive conclusions cannot be made given the small number of studies included, a lack of control for confounding and high heterogeneity. Furthermore, none of the studies analysed antibiotic exposure during childhood and adolescence, a time when individuals are most vulnerable to gut dysbiosis [8].

Although we found limited associations between antibiotic usage and rectal cancer across all age-groups, non-anti-anaerobic (i.e. exclusively anti-aerobic) antibiotics among the young age-group only were observed to increase risk of colon, rectal, proximal and distal colon cancer more than anti-anaerobic antibiotics. This conflicts with a case-control study of participants aged 40–70 years, which found anti-aerobic antibiotics to protect against distal colon and rectal cancer, whereas anti-anaerobic antibiotics increased risk of cancer—particularly in the proximal colon [4]. A further study [28] found both anti-aerobic and anti-anaerobic agents were associated with CRC, whereas another found just anti-anaerobic antibiotics increased risk [27]. However, sample sizes among our early-onset CRC cohort were small, especially when stratified into non-anti-anaerobic antibiotics and length of treatment. Furthermore, it may be clinically irrelevant whether anti-anaerobic or anti-aerobic antibiotics have a role in tumour formation, as most antibiotic drugs have dual anti-anaerobic and anti-aerobic activity.

Coinciding with existing studies, a very strong association was observed between antibiotic consumption and proximal colon cancer [4, 40, 49]; however, this was only observed in the early-onset CRC subgroup analysis, which had a small sample size of just 50 cases. With a greater microbial diversity and concentration of short-chain fatty acids, the proximal colon is more vulnerable to antibiotic exposure than the distal colon and rectum [49, 50]. Dysbiosis results in altered bacterial activity, fermentation and therefore colonic pH, in addition to interruption of protective colonic mucus leading to direct contact between the biofilm and epithelial cells, leading to chronic inflammation [12, 50, 51]. In all cohorts, there was limited evidence of a positive exposure-response relationship between cumulative antibiotic use and risk of CRC, with the exception of proximal colon cancer in the younger cohort. This supports previous literature suggesting risk increases after minimal antibiotic use [4], with risk not necessarily increasing with prolonged antibiotic exposure [7, 49].

Whether the observed relationship between antibiotics and CRC is causal remains uncertain. CRC is a complex, “heterogenous” disease with many underlying molecular mechanisms and risk factors [11]. Compared to later-onset disease, early-onset CRC has been described as a remarkably distinctive subset of disease [5, 6, 11]. Therefore, to compare our findings with previous studies, which have not considered the impact of age on CRC in addition to antibiotic exposure, may be inappropriate. If we were to disregard this fact, according to the Bradford Hill criteria [52], it is likely that a causal relationship may exist. Our findings indicate a strong association between antibiotic use and CRC, particularly with colon cancer. Our study is somewhat consistent with the literature, suggesting a relationship does exist—even if effect sizes vary. There is evidence of temporality in other studies [29], although we found no evidence of a biological gradient except in the case of early-onset proximal colon cancer. A causal relationship is plausible and coherent, and we can draw parallels with other commonly accepted phenomena—such as antibiotic-induced microbiome changes increasing risk of obesity, autoimmune disease and metabolic disorders [53–55], and the anticancer effects of a healthy microbiome [21]. However, the relationship is not particularly specific; with around 67 million courses of antibiotics prescribed each year in the USA to children aged less than 19 [18], exposure to antibiotics among the young is incredibly common. It is therefore hard to judge how many of these exposed individuals will potentially be diagnosed with early-onset CRC—a relatively rare disease outcome [9].

In our study, we observed more participants with CRC had rectal cancer in the younger rather than the older cohort. A study investigating USA early-onset CRC trends suggest rectal cancer incidence in the young is increasing more rapidly than colon cancer; by 2030, they predict incidence of colon and rectal cancer will increase by 90% and 124% among patients aged 20–34 years [56]. A possible association may exist between sexually transmitted infections and early-onset rectal cancer; [49] *Chlamydia* infections have malignant potential and secondary rectal infection is common [57, 58]. A review of clinical and molecular features of early-onset CRC suggests distal colon and rectal cancer are predominantly features of early-onset CRC, whereas proximal cancers tend to feature in later-onset disease [11]. Despite this, it is likely that sporadic early and later-onset CRC are otherwise indistinguishable in terms of genomics and biology [12]. The embryological origins of the proximal colon (midgut) and distal colon and rectum (hindgut) are different, as are biological features of cancers arising in these areas; proximal have more microsatellite instability, and distal more chromosomal instability [59]. Together with differences in the luminal contents and microbiome, it is biologically plausible that antibiotic consumption could influence the development of colonic and rectal cancer differentially by location.

There are multiple elements likely to be driving the increase in early-onset CRC including dietary factors—such as increased consumption of red and processed meat, monosodium glutamate, titanium dioxide and high-fructose corn syrup; obesity; stress; reduced exercise; and antibiotic consumption [5]. There is a scarcity of studies investigating early-life exposures and adult-onset cancers, although the aforementioned factors at interplay are known to have adverse effects on the microbiome. In addition, lifestyle changes occurring since the 1950s correlate with the increased rates of CRC, especially among the young [60]. Evidence of possible carcinogenic effects of antibiotics are limited [61], yet some antibiotics commonly up-regulate cyclooxygenase-2—a mechanism proven to promote development of CRC [62, 63]. Furthermore, it is the antibiotic-induced microbiome changes which disrupt immunostimulatory bacteria and give rise to pathogenic colonisation which is likely to be carcinogenic, rather than the actual medications themselves [21, 22].

This study has several strengths. PCCIUR is nationally representative, covering at least 15% of the Scottish general practice population [35]. Comprehensive linking of practice data to Scottish Cancer Registry data provides high coverage of CRC cases (given the relative rarity of early-onset CRC) and a relatively long exposure period. Thorough cleaning and validation of the data has minimised loss of prescription items due to transcription errors. This allowed accurate calculation of cumulative antibiotic exposure in primary care by class or spectrum of activity. In the UK, antibiotics can only be obtained with a medical prescription, and over-the-counter purchases are not possible. Although we could not access secondary care prescriptions, antibiotics commenced in hospital with long-term intent will appear in subsequent GP prescribing records. In our analyses we make a distinction between patients with early-onset CRC and later-onset CRC, and used sensitivity analysis to determine whether results changed when the age threshold used to define the two strata was altered.

Inevitably, this study also has its limitations. Given CRC in those aged less than 50 years is relatively rare, we had just 445 cases. Our sample size decreased further when we explored antibiotic spectrum of activity and specific tumour locations, and inevitably some analyses among patients under 50 years will be underpowered. Individuals with immunosuppressing conditions or diagnosed genetic predispositions to CRC were excluded; these make up a significant proportion of early-onset CRC patients, and the impact of antibiotic therapy in these groups will therefore not be measurable. Although we managed to exclude participants with genetic predispositions to CRC, the PCCIUR dataset does not provide information on a participants’ family history or dietary habits (BMI was only reported for ~33% of our sample). These both have a significant influence on CRC risk, for example the increased risk associated with obesity and CRC is well-known [64]. However the low numbers of cases among patients under 50 years where BMI was reported means that we cannot comment substantively on the nature of any association of BMI with CRC risk on the basis of our analyses. Long-term effects of exposure to antibiotics in childhood, when the gut microbiome is developing and potentially more vulnerable, are yet to be evaluated in terms of cancer risk and may be of clinical importance [65]. Unfortunately, lack of prescribing data in PCCIUR prior to 1993 means we are not able to explore whether initial age of exposure to antibiotics is associated with CRC risk.

Our dataset is suspectable to various biases associated with observational data, such as differential recall and reverse causation. The latter may be observed if patients presenting with stomach pain are initially diagnosed with gastrointestinal infection and prescribed antibiotics. Although we adjusted for use of medicines, smoking and alcohol use, residual confounding may be present (e.g. NSAID strength/duration of prescribing, units of alcohol consumed, number of pack years for smokers). There may be unmeasured confounding in the main analyses due to the inability to adjust for other relevant confounders not reported comprehensively in our data (e.g. BMI). Discrepencies in tumour location data may exist, with cases recorded as ‘colon’ rather than the sub-site within the colon. In addition, some patient groups will be missing from primary care records and cannot be accounted for, such as the homeless, private patients and prisoners. There will also be variability between prescribers regarding completeness of comorbidity recording. Data not captured by PCCIUR includes most secondary care prescriptions, private healthcare records, antibiotic prescriptions before 1993 (as these will not have been recorded electronically), and prescription adherence. These could be highly relevant to the study. Finally we cannot guarantee that patients adhered to their prescription medication, which seems likely to dilute any real associations, although studies suggest adherence to antibiotic therapy is high [66].

Our findings, showing no significant difference between the associations with early and later-onset disease, supporting recent evidence suggesting there are more similarities than differences between early and later-onset disease [12]. Therefore future studies to further elucidate any role of antibiotics in CRC genesis should be inclusive of all age-groups.

In conclusion, our findings suggest antibiotic exposure is associated with CRC genesis across all age-groups. It is possible that antibiotic exposure may be contributing to cases of CRC, potentially more so among the young. Our study raises the question whether antibiotic usage history should be included in the standardised proformas for referral from primary to secondary care. Further studies to confirm our findings and evaluate long-term effects of antibiotics on gut health are required, and increased awareness of the potential harms associated with antibiotic usage among clinicians and members of the public is necessary.

## Supplementary information

Supplementary Material

Academic Journals Reporting Checklist

STROBE Statement

## Data Availability

The datasets analysed in this study are not publicly available and were used under license. Requests for PCCIUR data should be directed in the first instance to Katie Wilde (Research Manager), email: k.wilde@abdn.ac.uk.

## Code availability

Standard statistical techniques were used to analyse the data, for which code can be obtained via the corresponding author. All statistical analyses were undertaken using Stata 15 [67]. Results were significant if they referred to statistical significance on a two-sided design-based test evaluated at the 5% level.
