# Supplementary material for: Oral antibiotic use and early-onset colorectal cancer: findings from a case-control study using a national clinical database
Source: Br J Cancer. 2021 Dec 17;126(6):957–67. doi: 10.1038/s41416-021-01665-7 (PMC8927122; doi:10.1038/s41416-021-01665-7)
Supplement: Supplementary file 1 — Supplementary Material [file 41416_2021_1665_MOESM1_ESM.docx]

**Supplementary Material**

Table S1 Read codes for case identification

Table S2 Medicines studied by class and therapeutic effect

Table S3 Characteristics of colon and rectal cancer cases and controls site

Table S4 Associations between duration of antibiotic use and colon/rectal cancer

Table S5 Associations between classes of antibiotics and colorectal cancer

Table S6 Associations between duration of antibiotic use and proximal/distal colon cancer

Table S7 Comparison of patients by recorded BMI

Table S8 Associations between antibiotic use and colorectal cancer, adjusting for comorbidities, medicine use and BMI

Table S9 Associations between antibiotic use and colorectal cancer, adjusting for comorbidities, medicine use, smoking status, alcohol use and BMI

**Table S1 Read codes for case identification**

| **Read code** | **Cancer type** | **Colon** | **Rectum** | **Proximal colon** | **Distal colon** |
| --- | --- | --- | --- | --- | --- |
| B13..00 | Malignant neoplasm of colon | X |  |  |  |
| B130.00 | Malignant neoplasm of hepatic flexure of colon | X |  | X |  |
| B131.00 | Malignant neoplasm of transverse colon | X |  | X |  |
| B132.00 | Malignant neoplasm of descending colon | X |  |  | X |
| B133.00 | Malignant neoplasm of sigmoid colon | X |  |  | X |
| B134.00 | Malignant neoplasm of caecum | X |  | X |  |
| B135.00 | Malignant neoplasm of appendix | X |  | X |  |
| B136.00 | Malignant neoplasm of ascending colon | X |  | X |  |
| B137.00 | Malignant neoplasm of splenic flexure of colon | X |  |  | X |
| B13y.00 | Malignant neoplasm of other specified sites of colon | X |  |  |  |
| B13z.00 | Malignant neoplasm of colon NOS | X |  |  |  |
| B140.00 | Malignant neoplasm of rectosigmoid junction |  | X |  |  |
| B141.00 | Malignant neoplasm of rectum |  | X |  |  |
| B14..00 | Malignant neoplasm of rectum, rectosigmoid junction and anus |  | X |  |  |
| B14y.00 | Malignant neoplasm of other sites of rectum, rectosigmoid junction and anus |  | X |  |  |
| B14z.00 | Malignant neoplasm of rectum, rectosigmoid junction and anus NOS |  | X |  |  |
| B13..00 | Malignant neoplasm of colon | X |  |  |  |
| B130.00 | Malignant neoplasm of hepatic flexure of colon | X |  | X |  |
| B131.00 | Malignant neoplasm of transverse colon | X |  | X |  |

**Table S2 Medicines studied by class and therapeutic effect on gut microbiome**

| **Class** | **Medicine** | **Spectrum of activity** | **Primary clinical therapeutic use** | **Other antimicrobial activity*** |
| --- | --- | --- | --- | --- |
| Penicillins | amoxicillin | Broad | Anti-anaerobic/anti-aerobic | N/A |
|  | ampicillin | Broad | Anti-anaerobic/anti-aerobic | N/A |
|  | flucloxacillin | Broad | Anti-aerobic | Anti-anaerobic |
|  | phenoxymethylpenicillin | Broad | Anti-anaerobic/anti-aerobic | N/A |
|  | cloxacillin | Broad | Anti-aerobic | Anti-anaerobic |
|  | benzylpenicillin | Broad | Anti-anaerobic/anti-aerobic | N/A |
|  | pivampicillin | Broad | Anti-anaerobic/anti-aerobic | N/A |
|  | pivmecillinam | Broad | Anti-anaerobic | N/A |
|  | pivampicillin/pivmecillinam | Broad | Anti-anaerobic/anti-aerobic | N/A |
|  | flucloxacillin & ampicillin  (co-fluampicil) | Broad | Anti-anaerobic/anti-aerobic | N/A |
| Tetracyclines | tetracycline | Broad | Anti-anaerobic/anti-aerobic | N/A |
|  | oxytetracycline | Broad | Anti-anaerobic/anti-aerobic | N/A |
|  | doxycycline | Broad | Anti-anaerobic/anti-aerobic | N/A |
|  | minocycline | Broad | Anti-anaerobic/anti-aerobic | N/A |
|  | lymecycline | Broad | Anti-anaerobic/anti-aerobic | N/A |
|  | demeclocycline | Broad | Anti-anaerobic/anti-aerobic | N/A |
|  | chlortetracycline | Broad | Anti-anaerobic/anti-aerobic | N/A |
|  | tetracycline hydrochloride,  chlortetracycline hydrochloride & demeclocycline hydrochloride | Broad | Anti-anaerobic/anti-aerobic | N/A |
| Cephalosporins | cefalexin | Broad | Anti-aerobic | Partial anti-anaerobic |
|  | cefuroxime | Broad | Anti-aerobic | Partial anti-anaerobic |
|  | cefradine | Broad | Anti-aerobic | Partial anti-anaerobic |
|  | cefixime | Broad | Anti-aerobic | Partial anti-anaerobic |
| Macrolides | erythromycin | Broad | Anti-aerobic | Partial anti-anaerobic |
|  | clarithromycin | Broad | Anti-aerobic | Partial anti-anaerobic |
|  | azithromycin | Broad | Anti-aerobic | Partial anti-anaerobic |
| Quinolones (& nalidixic acid) | ciprofloxacin | Broad | Anti-aerobic | Partial anti-anaerobic |
|  | ofloxacin | Broad | Anti-aerobic | Partial anti-anaerobic |
|  | norfloxacin | Broad | Anti-aerobic | N/A |
|  | levofloxacin | Broad | Anti-aerobic | Anti-anaerobic |
|  | moxifloxacin | Broad | Anti-anaerobic/anti-aerobic | N/A |
|  | nalidixic acid | Narrow | Anti-aerobic | Partial anti-anaerobic |
| Sulpha & trimethoprim | trimethoprim | Narrow | Anti-aerobic | N/A |
|  | sulfametopyrazine | Narrow | Anti-aerobic | N/A |
|  | sulfadiazine | Narrow | Anti-aerobic | N/A |
|  | trimethoprim/sulfamethoxazole  (co-trimoxazole) | Narrow | Anti-aerobic | N/A |
| Lincomycins | clindamycin | Broad | Anti-anaerobic/anti-aerobic | N/A |
| Novel | fosfomycin | Narrow | Anti-aerobic | Anti-anaerobic |
|  | nitrofurantoin | Narrow | Anti-aerobic | Anti-anaerobic |
| Nitroimidazoles | metronidazole | Narrow | Anti-anaerobic | N/A |
|  | tinidazole | Narrow | Anti-anaerobic | N/A |
| Glycopeptides | vancomycin | Broad | Anti-anaerobic | Anti-aerobic |
| Aminoglycosides | neomycin | Broad | Anti-aerobic | N/A |
|  | tobramycin | Narrow | Anti-aerobic | N/A |
| **Class** | **Medicine** | **Spectrum of activity** | **Primary clinical therapeutic use** | **Other antimicrobial activity*** |
| Antimycobacterials | isoniazid | Narrow | Anti-aerobic | N/A |
|  | rifampicin | Narrow | Anti-aerobic | N/A |
|  | rifabutin | Narrow | Anti-aerobic | N/A |
|  | rifampicin with isoniazid | Narrow | Anti-aerobic | N/A |

N/A: not applicable; * potential off-target antimicrobial activity

**Table S3 Characteristics of colon and rectal cancer cases and controls**

| **Variable** | **Category** | **Colon** | | | | **Rectum** | | | |
| --- | --- | --- | --- | --- | --- | --- | --- | --- | --- |
|  |  | **<50 years** | | **>=50 years** | | **<50 years** | | **>=50 years** | |
|  |  | **Cases**  ***N* (%)** | **Controls**  ***N* (%)** | **Cases**  ***N* (%)** | **Controls**  ***N* (%)** | **Cases**  ***N* (%)** | **Controls**  ***N* (%)** | **Cases**  ***N* (%)** | **Controls**  ***N* (%)** |
| Number of patients |  | 289 | 1,217 | 4,992 | 19,072 | 156 | 673 | 2,466 | 9,456 |
| Length of exposure period (years): median (IQR) |  | 7·0 (4·7,9·5) | 6·8 (4·6,9·4) | 8·1 (5·5,11·1) | 7·9 (5·4,10·9) | 7·0 (5·1,9·0) | 7·0 (5·1,9·0) | 7·7 (5·2,10·6) | 7·6 (5·1,10·5) |
| Year of diagnosis/index date: median (IQR) |  | 2005 (2002,2007) | 2004 (2002,2007) | 2005 (2002,2007) | 2005 (2002,2007) | 2003 (2001,2007) | 2003 (2001,2007) | 2004 (2002,2006) | 2004 (2001,2006) |
| Age at diagnosis/index date: median (IQR) |  | 44 (40,47) | 42 (38,45) | 72 (64,79) | 69 (61,77) | 45 (42,47) | 42 (39,45) | 70 (63,77) | 68 (60,76) |
| Deprivation quintile | 1 (least deprived) | 28 (9·7) | 116 (9·5) | 594 (11·9) | 2,314 (12·1) | 20 (12·8) | 91 (13·5) | 294 (11·9) | 1,152 (12·2) |
|  | 2 | 90 (31·1) | 370 (30·4) | 1,403 (28·1) | 5,396 (28·3) | 35 (22·4) | 155 (23·0) | 674 (27·3) | 2,609 (27·6) |
|  | 3 | 23 (8·0) | 100 (8·2) | 474 (9·5) | 1,828 (9·6) | 15 (9·6) | 59 (8·8) | 225 (9·1) | 890 (9·4) |
|  | 4 | 67 (23·2) | 290 (23·8) | 1,259 (25·2) | 4,784 (25·1) | 40 (25·6) | 163 (24·2) | 642 (26·0) | 2,408 (25·5) |
|  | 5 (most deprived) | 80 (27·7) | 339 (27·9) | 1,256 (25·2) | 4,730 (24·8) | 46 (29·5) | 205 (30·5) | 630 (25·5) | 2,392 (25·3) |
|  | Missing | <=5 (<=1·7) | <=5 (<=0·4) | 6 (0·1) | 20 (0·1) | 0 (0·0) | 0 (0·0) | <=5 (<=0·2) | <=5 (<=0·1) |
| Gender | Male | 146 (50·5) | 625 (51·4) | 2,648 (53·0) | 10,164 (53·3) | 84 (53·9) | 372 (55·3) | 1,505 (61·0) | 5,834 (61·7) |
|  | Female | 143 (49·5) | 592 (48·6) | 2,344 (47·0) | 88,908 (46·7) | 72 (46·2) | 301 (44·7) | 961 (39·0) | 3,622 (38·3) |
| Smoking status^†^ | never smoked | 113 (39·1) | 444 (36·5) | 1,921 (38·5) | 6,964 (36·5) | 52 (33·3) | 216 (32·1) | 855 (34·7) | 3,271 (34·6) |
|  | ex-smoker | 35 (12·1) | 93 (7·6) | 1,189 (23·8) | 3,605 (18·9) | 13 (8·3) | 45 (6·7) | 538 (21·8) | 1,771 (18·7) |
|  | current smoker | 59 (20·4) | 290 (23·8) | 749 (15·0) | 3,549 (18·6) | 43 (27·6) | 156 (23·2) | 444 (18·00 | 1,826 (19·3) |
|  | missing | 82 (28·4) | 390 (32·1) | 1,133 (22·7) | 4,954 (26·0) | 48 (30·8) | 256 (38·0) | 629 (25·5) | 2,588 (27·4) |

| **Variable** | **Category** | **Colon** | | | | **Rectum** | | | |
| --- | --- | --- | --- | --- | --- | --- | --- | --- | --- |
|  |  | **<50 years** | | **>=50 years** | | **<50 years** | | **>=50 years** | |
|  |  | **Cases**  ***N* (%)** | **Controls**  ***N* (%)** | **Cases**  ***N* (%)** | **Controls**  ***N* (%)** | **Cases**  ***N* (%)** | **Controls**  ***N* (%)** | **Cases**  ***N* (%)** | **Controls**  ***N* (%)** |
| Alcohol consumption^†^ | non-drinker | 18 (6·2) | 108 (8·9) | 763 (15·3) | 2,747 (14·4) | 18 (11·5) | 57 (8·5) | 336 (13·6) | 1,309 (13·84) |
|  | light/moderate | 162 (56·1) | 561 (4601) | 2,435 (48·8) | 8,806 (46·2) | 76 (48·7) | 285 (42·4) | 1,128 (45·7) | 4,247 (44·9) |
|  | heavy drinker | 7 (2·4) | 40 (3·3) | 192 (3·9) | 679 (3·6) | 6 (3·9) | 16 (2·4) | 139 (5·6) | 407 (4·3) |
|  | missing | 102 (35·3) | 508 (41·7) | 1,602 (32·1) | 6,840 (35·9) | 56 (36·9) | 315 (46·8) | 863 (35·0) | 3,493 (36·9) |
| Comorbidities diagnosed prior to or during the exposure period |  |  |  |  |  |  |  |  |  |
| Diabetes |  | <=5 (<=1·7) | 24 (2·0) | 569 (11·9) | 1,536 (8·1) | <=5 (<=3·2) | 14 (2·1) | 250 (10·1) | 798 (8·4) |
| Myocardial infarction |  | 0 (0·0) | 6 (0·5) | 387 (7·8) | 1,277 (6·7) | 0 (0·0) | <=5 (<=0·7) | 157 (6·4) | 637 (6·7) |
| Coronary heart disease |  | <=5 (<=1·7) | 11 (0·9) | 954 (19·1) | 3,121 (16·4) | <=5 (<=3·2) | <=5 (<=0·7) | 383 (15·5) | 1,467 (15·5) |
| Heart failure |  | <=5 (<=1·7) | <=5 (<=0·4) | 207 (4·2) | 653 (3·4) | 0 (0·0) | 0 (0·0) | 92 (3·7) | 301 (3·2) |
| Peripheral vascular disease |  | <=5 (<=1·7) | 6 (0·5) | 230 (4·6) | 852 (4·5) | <=5 (<=3·2) | <=5 (<=0·7) | 125 (5·1) | 402 (4·3) |
| Dementia |  | 0 (0·0) | 0 (0·0) | 38 (0·8) | 326 (1·7) | 0 (0·0) | 0 (0·0) | 19 (0·8) | 118 (1·3) |
| Cerebrovascular disease |  | <=5 (<=1·7) | 8 (0·7) | 412 (8·3) | 1,442 (7·6) | <=5 (<=3·2) | <=5 (<=0·7) | 181 (7·3) | 670 (7·1) |
| Chronic obstructive pulmonary disease |  | <=5 (<=1·7) | 17 (1·4) | 329 (6·6) | 1,181 (6·2) | <=5 (<=3·2) | 14 (2·1) | 169 (6·9) | 531 (5·6) |
| Osteoporosis |  | 0 (0·0) | 0 (0·0) | 140 (2·8) | 540 (2·8) | 0 (0·0) | 0 (0·0) | 38 (1·5) | 210 (2·2) |
| Renal disease |  | <=5 (<=1·7) | <=5 (<=0·4) | 210 (4·2) | 6354 (3·3) | <=5 (<=3·2) | <=5 (<=0·7) | 101 (4·1) | 267 (2·8) |
| Liver disease |  | 0 (0·0) | <=5 (<=0·4) | 33 (0·7) | 128 (0·7) | 0 (0·0) | <=5 (<=0·7) | 19 (·8) | 71 (0·8) |
| Hemiplegia/paraplegia |  | <=5 (<=1·7) | <=5 (<=0·4) | 33 (0·7) | 98 (0·5) | 0 (0·0) | <=5 (<=0·7) | 13 (0·5) | 64 (0·7) |
| Gallstones |  | <=5 (<=1·7) | 10 (0·8) | 362 (7·3) | 1,053 (5·5) | <=5 (<=3·2) | 13 (1·9) | 121 (4·9) | 472 (5·0) |
| Acromegaly |  | 0 (0·0) | 0 (0·0) | 0 (0·0) | <=5 (<=0·1) | <=5 (<=3·2) | 0 (0·00 | 0 (0·0) | <=5 (<=0·1) |
| Medication use during exposure period |  |  |  |  |  |  |  |  |  |
| Low dose aspirin |  | 9 (3·1) | 16 (1·3) | 1,510 (32·3) | 4,997 (26·2) | <=5 (<=3·2) | 17 (2·5) | 647 (26·2) | 2,452 (24·9) |
| NSAIDs |  | 82 (28·4) | 316 (26·0) | 1,892 (37·9) | 7,245 (38·0) | 46 (29·5) | 166 (24·7) | 862 (35·0) | 3,401 (36·0) |

Abbreviations: IQR: inter-quartile range; NSAIDS: non-steroidal anti-inflammatory drugs

^†^ most recent record in patient’s clinical history prior to one-year lag

**Table S4 Associations between duration of antibiotic use and colon/rectal cancer**

|  |  |  | **<50 years** | | | | | **>=50 years** | | | | |
| --- | --- | --- | --- | --- | --- | --- | --- | --- | --- | --- | --- | --- |
| **Site** | **Medicine** | **Cumulative exposure** | **Cases**  ***N* (%)** | **Controls**  ***N* (%)** | **Adjusted ‡OR (95%CI)** | ***P-*value** | ***P-*trend** | **Cases**  ***N* (%)** | **Controls**  ***N* (%)** | **Adjusted ‡OR (95%CI)** | ***P-*value** | ***P-*trend** |
| **colon** | **any antibiotic use** | **0 days** | 148 (51·2%) | 725 (59·6%) | 1·00 |  | 0·177 | 2,570 (51·5%) | 10,297 (54·0%) | 1·00 |  | 0·082 |
|  |  | **1-15 days** | 87 (30·1%) | 281 (23·1%) | 1·55 (1·09,2·20) | 0·015 |  | 1,363 (27·3%) | 4,959 (26·0%) | 1·09 (1·00,1·19) | 0·039 |  |
|  |  | **16-60 days** | 44 (15·2%) | 162 (13·3%) | 1·46 (0·91,2·33) | 0·113 |  | 832 (16·7%) | 3,027 (15·9%) | 1·08 (0·97,1·20) | 0·162 |  |
|  |  | **>60 days** | 10 (3·5%) | 49 (4·0%) | 0·99 (0·45,2·19) | 0·984 |  | 227 (4·6%) | 789 (4·1%) | 1·11 (0·93,1·31) | 0·244 |  |
|  | **anti-anaerobic activity *** | **0 days** | 182 (63·0%) | 831 (68·3%) | 1·00 |  | 0·386 | 3,165 (63·4%) | 12,478 (65·4%) | 1·00 |  | 0·105 |
|  |  | **1-15 days** | 74 (25·6%) | 251 (20·6%) | 1·44 (1·01,2·05) | 0·045 |  | 1,281 (25·7%) | 4,624 (24·2%) | 1·08 (0·99,1·17) | 0·077 |  |
|  |  | **16-60 days** | 28 (9·7%) | 106 (8·7%) | 1·20 (0·71,2·03) | 0·507 |  | 452 (9·1%) | 1,618 (8·5%) | 1·09 (0·96,1·23) | 0·199 |  |
|  |  | **>60 days** | <=5 (<=1·7%) | 29 (2·4%) | 0·79 (0·27,2·27) | 0·659 |  | 94 (1·9%) | 352 (1·9%) | 1·06 (0·83,1·35) | 0·641 |  |
|  | **non anti-anaerobic activity *** | **0 days** | 198 (68·5%) | 914 (75·1%) | 1·00 |  | 0·108 | 3,407 (68·3%) | 13,331 (69·9%) | 1·00 |  | 0·945 |
|  |  | **1-15 days** | 72 (24·9%) | 236 (19·4%) | 1·39 (0·97,2·00) | 0·070 |  | 1,137 (22·8%) | 4,088 (21·4%) | 1·05 (0·97,1·14) | 0·251 |  |
|  |  | **16-60 days** | 15 (5·2%) | 56 (4·6%) | 1·07 (0·54,2·14) | 0·846 |  | 369 (7·4%) | 1,397 (7·3%) | 0·94 (0·83,1·08) | 0·403 |  |
|  |  | **>60 days** | <=5 (<=1·7%) | 11 (0·9%) | 2·52 (0·72,8·74) | 0·146 |  | 79 (1·6%) | 256 (1·3%) | 1·05 (0·80,1·37) | 0·726 |  |
|  |  |  |  |  |  |  |  |  |  |  |  |  |
| **rectum** | **any antibiotic** | **0 days** | 87 (55·8%) | 408 (60·6%) | 1·00 |  | 0·233 | 1,392 (56·5%) | 5,488 (58·0%) | 1·00 |  | 0·656 |
|  |  | **1-15 days** | 37 (23·7%) | 159 (23·6%) | 0·98 (0·59,1·63) | 0·935 |  | 625 (25·3%) | 2,302 (24·3%) | 1·08 (0·96,1·22) | 0·208 |  |
|  |  | **16-60 days** | 24 (15·4%) | 74 (11·0%) | 1·87 (0·97,3·60) | 0·061 |  | 367 (14·9%) | 1,320 (14·0%) | 1·08 (0·93,1·26) | 0·306 |  |
|  |  | **>60 days** | 8 (5·1%) | 32 (4·8%) | 1·20 (0·47,3·02) | 0·706 |  | 82 (3·3%) | 346 (3·7%) | 0·91 (0·69,1·18) | 0·463 |  |
|  | **anti-anaerobic activity *** | **0 days** | 100 (64·1%) | 457 (67·9%) | 1·00 |  | 0·865 | 1,663 (67·4%) | 6,430 (68·0%) | 1·00 |  | 0·998 |
|  |  | **1-15 days** | 38 (24·4%) | 136 (20·2%) | 1·18 (0·71,1·95) | 0·517 |  | 564 (22·9%) | 2,153 (22·8%) | 0·99 (0·88,1·12) | 0·883 |  |
|  |  | **16-60 days** | 13 (8·3%) | 61 (9·1%) | 0·91 (0·42,1·98) | 0·815 |  | 199 (8·1%) | 708 (7·5%) | 1·05 (0·87,1·26) | 0·634 |  |
|  |  | **>60 days** | <=5 (<=3·2%) | 19 (2·8%) | 1·11 (0·36,3·42) | 0·852 |  | 40 (1·6%) | 165 (1·7%) | 0·91 (0·63,1·32) | 0·623 |  |
|  | **non anti-anaerobic activity *** | **0 days** | 112 (71·8%) | 533 (79·2%) | 1·00 |  | 0·115 | 1,782 (72·3%) | 6,957 (73·6%) | 1·00 |  | 0·934 |
|  |  | **1-15 days** | 34 (21·8%) | 101 (15·0%) | 1·78 (1·06,2·99) | 0·029 |  | 513 (20·8%) | 1,812 (19·2%) | 1·08 (0·96,1·22) | 0·215 |  |
|  |  | **16-60 days** | 8 (5·1%) | 29 (4·3%) | 1·68 (0·67,4·17) | 0·266 |  | 145 (5·9%) | 569 (6·0%) | 0·97 (0·79,1·20) | 0·788 |  |
|  |  | **>60 days** | <=5 (<=3·2%) | 10 (1·5%) | 0·93 (0·17,5·00) | 0·929 |  | 26 (1·1%) | 118 (1·3%) | 0·80 (0·52,1·26) | 0·340 |  |

Abbreviations: OR: odds ratio, CI confidence interval; * primary clinical therapeutic effect on gut microbe; ‡ adjusted for diabetes, myocardial infarction, coronary heart disease, heart failure, peripheral vascular disease, dementia, cerebrovascular disease, chronic obstructive pulmonary disease, osteoporosis, renal disease, liver disease, hemiplegia/paraplegia, gallstones, acromegaly, low dose aspirin and NSAIDs

**Table S5 Associations between classes of antibiotics and colorectal cancer**

|  |  | **<50 years** | | | | **>=50 years** | | | |  |
| --- | --- | --- | --- | --- | --- | --- | --- | --- | --- | --- |
| **Site** | **Medicine** | **Cases**  **n (%)** | **Controls**  **n (%)** | **Adjusted‡ OR (95%CI)** | **Adjusted‡ p-value** | **Cases**  **n (%)** | **Controls**  **n (%)** | **Adjusted‡ OR (95%CI)** | **Adjusted‡ p-value** | **Interaction test p-value** |
| **colon** | **cephalosporins** | 19 (6·6%) | 64 (5·3%) | 1·37 (0·74,2·52) | 0·313 | 417 (8·6%) | 1,463 (7·7%) | 1·02 (0·90,1·16) | 0·703 | 0·362 |
|  | **macrolides** | 33 (11·4%) | 125 (10·3%) | 0·97 (0·60,1·56) | 0·903 | 575 (11·5%) | 2,030 (10·6%) | 1·07 (0·96,1·19) | 0·225 | 0·700 |
|  | **penicillins** | 105 (36·3%) | 369 (30·3%) | 1·39 (1·00,1·94) | 0·047 | 1,884 (37·7%) | 6,742 (35·6%) | 1·09 (1·01,1·18) | 0·031 | 0·151 |
|  | **quinolones & nalidixic acid** | 14 (4·8%) | 38 (3·1%) | 1·79 (0·85,3·79) | 0·125 | 258 (5·2%) | 1,044 (5·5%) | 0·87 (0·75,1·01) | 0·067 | 0·062 |
|  | **sulpha & trimethoprim** | 26 (9·0%) | 87 (7·2%) | 1·14 (0·69,1·88) | 0·618 | 539 (10·8%) | 1,937 (10·2%) | 0·97 (0·87,1·09) | 0·600 | 0·548 |
|  | **tetracyclines** | 27 (9·3%) | 103 (8·5%) | 1·09 (0·67,1·78) | 0·716 | 360 (7·2%) | 1,362 (7·1%) | 1·01 (0·89,1·16) | 0·833 | 0·766 |
|  | **other** | 13 (4·5%) | 48 (3·9%) | 1·09 (0·55,2·14) | 0·801 | 192 (3·9%) | 704 (3·7%) | 0·99 (0·83,1·17) | 0·865 | 0·775 |
| **rectrum** | **cephalosporins** | 9(5·77%) | 27(4·01%) | 1·68(0·68,4·14) | 0·263 | 158(6·41%) | 622(6·58%) | 0·94(0·77,1·15) | 0·567 | 0·263 |
|  | **macrolides** | 19(12·18%) | 71(10·55%) | 1·37(0·73,2·54) | 0·326 | 234(9·49%) | 862(9·12%) | 1·03(0·87,1·21) | 0·740 | 0·388 |
|  | **penicillins** | 55(35·26%) | 214(31·80%) | 1·05(0·66,1·66) | 0·840 | 819(33·21%) | 3,058(32·34%) | 1·01(0·91,1·13) | 0·813 | 0·888 |
|  | **quinolones & nalidixic acid** | 6(3·85%) | 20(2·97%) | 1·71(0·56,5·22) | 0·349 | 116(4·70%) | 432(4·57%) | 1·01(0·81,1·26) | 0·948 | 0·365 |
|  | **sulpha & trimethoprim** | 12(7·69%) | 36(5·35%) | 1·83(0·83,4·01) | 0·133 | 216(8·76%) | 781(8·26%) | 1·03(0·86,1·22) | 0·776 | 0·160 |
|  | **tetracyclines** | 12(7·69%) | 60(8·92%) | 0·77(0·37,1·62) | 0·493 | 150(6·08%) | 638(6·75%) | 0·89(0·73,1·08) | 0·242 | 0·716 |
|  | **other** | 8(5·13%) | 29(4·31%) | 1·20(0·49,2·96) | 0·692 | 79(3·20%) | 332(3·51%) | 0·87(0·67,1·13) | 0·299 | 0·265 |

Abbreviations: OR: odds ratio, CI confidence interval; ‡ adjusted for diabetes, myocardial infarction, coronary heart disease, heart failure, peripheral vascular disease, dementia, cerebrovascular disease, chronic obstructive pulmonary disease, osteoporosis, renal disease, liver disease, hemiplegia/paraplegia, gallstones, acromegaly, low dose aspirin and NSAIDs

**Table S6 Associations between duration of antibiotic use and proximal colon/distal colon cancer**

|  |  |  | **<50 years** | | | | | **>=50 years** | | | | |
| --- | --- | --- | --- | --- | --- | --- | --- | --- | --- | --- | --- | --- |
| **Site** | **Medicine** | **Cumulative exposure** | **Cases**  ***N* (%)** | **Controls**  ***N* (%)** | **Adjusted ‡OR (95%CI)** | ***P-*value** | ***P-*trend** | **Cases**  ***N* (%)** | **Controls**  ***N* (%)** | **Adjusted ‡OR (95%CI)** | ***P-*value** | ***P-*trend** |
| **proximal colon** | **any antibiotic use** | **0 days** | 19 (38·0%) | 125 (59·0%) | 1·00 |  | 0·004 | 365 (57·3%) | 1,370 (56·7%) | 1·00 |  | 0·381 |
|  |  | **1-15 days** | 20 (40·0%) | 52 (24·5%) | 3·71 (1·51,9·12) | 0·004 |  | 154 (24·2%) | 565 (23·4%) | 0·96 (0·75,1·22) | 0·725 |  |
|  |  | **16-60 days** | 7 (14·0%) | 27 (12·7%) | 3·13 (0·91,10·80) | 0·071 |  | 90 (14·1%) | 414 (17·1%) | 0·72 (0·53,0·98) | 0·036 |  |
|  |  | **>60 days** | <=5 (<=10·0%) | 8 (3·8%) | 8·62 (1·73,42·90) | 0·009 |  | 28 (4·4%) | 69 (2·9%) | 1·33 (0·80,2·20) | 0·268 |  |
|  | **anti-anaerobic activity *** | **0 days** | 28 (56·0%) | 148 (69·8%) | 1·00 |  | 0·096 | 429 (67·4%) | 1,674 (69·2%) | 1·00 |  | 0·414 |
|  |  | **1-15 days** | 16 (32·0%) | 42 (19·8%) | 2·39 (1·01,5·66) | 0·048 |  | 147 (23·1%) | 524 (21·7%) | 1·07 (0·84,1·37) | 0·586 |  |
|  |  | **16-60 days** | <=5 (<=10·0%) | 17 (8·0%) | 1·40 (0·38,5·12) | 0·611 |  | 51 (8·0%) | 199 (8·2%) | 0·99 (0·69,1·44) | 0·973 |  |
|  |  | **>60 days** | <=5 (<=10·0%) | <=5 (<=2·4%) | 3·54 (0·50,24·98) | 0·204 |  | 10 (1·6%) | 21 (0·9%) | 1·91 (0·86,4·26) | 0·112 |  |
|  | **non anti-anaerobic activity *** | **0 days** | 31 (62·0%) | 159 (75·0%) | 1·00 |  | 0·003 | 458 (71·9%) | 1,677 (69·4%) | 1·00 |  | 0·094 |
|  |  | **1-15 days** | 13 (26·0%) | 39 (18·4%) | 2·59 (1·05,6·39) | 0·039 |  | 120 (18·8%) | 520 (21·5%) | 0·78 (0·61,1·00) | 0·052 |  |
|  |  | **16-60 days** | <=5 (<=10·0%) | 13 (6·1%) | 2·97 (0·59,15·00) | 0·187 |  | 50 (7·9%) | 196 (8·1%) | 0·75 (0·52,1·10) | 0·142 |  |
|  |  | **>60 days** | <=5 (<=10·0%) | <=5 (<=2·4%) |  |  |  | 9 (1·4%) | 25 (1·0%) | 1·03 (0·46,2·29) | 0·950 |  |
| **distal colon** | **any antibiotic**  **use** | **0 days** | 16 (44·4%) | 90 (60·0%) | 1·00 |  | 0·670 | 270 (52·4%) | 1,132 (55·8%) | 1·00 |  | 0·433 |
|  |  | **1-15 days** | 18 (50·0%) | 30 (20·0%) | 5·06 (1·40,18·29) | 0·013 |  | 155 (30·1%) | 508 (25·1%) | 1·30 (0·99,1·70) | 0·058 |  |
|  |  | **16-60 days** | <=5 (<=13·9%) | 21 (14·0%) | 1·05 (0·13,8·29) | 0·960 |  | 74 (14·4%) | 297 (14·6%) | 0·97 (0·68,1·38) | 0·847 |  |
|  |  | **>60 days** | 0 (0·0%) | 9 (6·0%) |  |  |  | 16 (3·1%) | 91 (4·5%) | 0·62 (0·34,1·14) | 0·125 |  |
|  | **anti-anaerobic activity *** | **0 days** | 24 (66·7%) | 99 (66·0%) | 1·00 |  | 0193 | 337 (65·4%) | 1,353 (66·7%) | 1·00 |  | 0·460 |
|  |  | **1-15 days** | 11 (30·6%) | 27 (18·0%) | 1·91 (0·58,6·26) | 0·288 |  | 129 (25·1%) | 461 (22·7%) | 1·07 (0·82,1·40) | 0·607 |  |
|  |  | **16-60 days** | <=5 (<=13·9%) | 21 (14·0%) | 0·11 (0·01,1·41) | 0·089 |  | 43 (8·4%) | 176 (8·7%) | 0·88 (0·58,1·33) | 0·543 |  |
|  |  | **>60 days** | 0 (0·0%) | <=5 (<=3·3%) |  |  |  | 6 (1·2%) | 38 (1·9%) | 0·60 (0·24,1·49) | 0·272 |  |
|  | **non anti-anaerobic activity *** | **0 days** | 20 (55·6%) | 119 (79·3%) | 1·00 |  | 0·110 | 363 (70·5%) | 1,450 (71·5%) | 1·00 |  | 0·331 |
|  |  | **1-15 days** | 16 (44·4%) | 21 (14·0%) | 10·41 (2·81,38·65) | <0·001 |  | 120 (23·3%) | 414 (20·4%) | 1·13 (0·87,1·48) | 0·360 |  |
|  |  | **16-60 days** | 0 (0·0%) | 7 (4·7%) |  |  |  | 24 (4·7%) | 138 (6·8%) | 0·58 (0·34,0·96) | 0·035 |  |
|  |  | **>60 days** | 0 (0·0%) | <=5 (<=3·3%) |  |  |  | 8 (1·6%) | 26 (1·3%) | 0·93 (0·39,2·21) | 0·863 |  |

Abbreviations: OR: odds ratio, CI confidence interval; * primary clinical therapeutic effect on gut microbe; ‡ adjusted for diabetes, myocardial infarction, coronary heart disease, heart failure, peripheral vascular disease, dementia, cerebrovascular disease, chronic obstructive pulmonary disease, osteoporosis, renal disease, liver disease, hemiplegia/paraplegia, gallstones, acromegaly, low dose aspirin and NSAIDs.

**Table S7 Comparison of patients by recorded BMI**

| **Variable** | | **BMI recorded** | **BMI not recorded** |
| --- | --- | --- | --- |
|  | | **n (%)** | **n (%)** |
| **Number of patients** | | 12,657 (33·0%) | 25,664 (66·7%) |
| **Length of exposure period (years): median (IQR)** | | 9·9 (7·1,12·5) | 7·0 (5·0,9·6) |
| **Year of diagnosis/index date: median (IQR)** | | 2006 (2005,2008) | 2003 (2001,2005) |
| **Age at diagnosis/index date: median (IQR)** | | 70 (61,78) | 67 (59,76) |
| **Deprivation quintile** | **1 (least deprived)** | 1,203 (9·5%) | 3,406 (13·3%) |
|  | **2** | 3,663 (28·9%) | 7,069 (27·5%) |
|  | **3** | 1,228 (9·7%) | 2,386 (9·3%) |
|  | **4** | 3,325 (26·3%) | 6,328 (24·7%) |
|  | **5 (most deprived)** | 3,217 (25·4%) | 6,461 (25·2%) |
|  | **Missing** | 21 (0·2%) | 14 (0·1%) |
| **Gender** | **male** | 7,428 (57·3%) | 14,130 (55·1%) |
|  | **female** | 5,409 (42·7%) | 11,534 (44·9%) |
| **Smoking status^†^** | **never smoked** | 5,708 (45·1%) | 8,128 (31·7%) |
|  | **ex-smoker** | 3,701 (29·2%) | 3,588 (14·0%) |
|  | **current smoker** | 2,579 (20·4%) | 4,537 (17·7%) |
|  | **missing** | 669 (5·3%) | 9,411 (36·7%) |
| **Alcohol consumption^†^** | **non-drinker** | 2,337 (18·5%) | 3,019 (11·8%) |
|  | **light/moderate** | 7,965 (62·9%) | 9,735 (37·9%) |
|  | **heavy drinker** | 720 (5·7%) | 766 (3·0%) |
|  | **missing** | 1,635 (12·9%) | 12,144 (47·3%) |
| **Body Mass Index: median (IQR)** |  | 26·7 (23·9,29·9) | - |
| **Body Mass Index** | **<25** | 4,310 (34·1%) | - |
|  | **>=25 & <30 (overweight)** | 5,188 (41·0%) | - |
|  | **>=30 (obese)** | 3,159 (25·0%) | - |
|  | |  | |
| **Comorbidities diagnosed prior to or during the exposure period** | |  | |
| **Diabetes** | | 2,097 (16·6%) | 1,128 (4·4%) |
| **Myocardial infarction** | | 1,236 (9·8%) | 1,232 (4·8%) |
| **Coronary heart disease** | | 2,817 (22·3%) | 3,126 (12·2%) |
| **Heart failure** | | 511 (4·0%) | 747 (2·9%) |
| **Peripheral vascular disease** | | 711 (5·6%) | 910 (3·6%) |
| **Dementia** | | 155 (1·2%) | 346 (1·4%) |
| **Cerebrovascular disease** | | 1,114 (8·8%) | 1,602 (6·2%) |
| **Chronic obstructive pulmonary disease** | | 836 (6·6%) | 1,412 (5·5%) |
| **Osteoporosis** | | 377 (3·0%) | 551 (2·2%) |
| **Renal disease** | | 810 (6·4%) | 411 (1·6%) |
| **Liver disease** | | 115 (0·9%) | 139 (0·5%) |
| **Hemiplegia/paraplegia** | | 80 (0·6%) | 134 (0·5%) |
| **Gallstones** | | 855 (6·8%) | 1,184 (4·6%) |
| **Acromegaly** | | <=5 (<=0·5%) | <=5 (<=0·0%) |
| **Medication use during exposure period** | |  |  |
| **Low dose aspirin** | | 4,789 (37·8%) | 4,761 (18·6%) |
| **NSAIDs** | | 5,876 (46·4%) | 8,134 (31·7%) |

Abbreviations: BMI: body mass index, IQR: inter-quartile range; NSAIDs: non-steroidal anti-inflammatory drugs

† most recent record in patient’s clinical history prior to one-year lag

**Table S8 Associations between antibiotic use and colorectal cancer, adjusting for comorbidities, medicine use and BMI**

|  |  | **<50 years** | | | | **>=50 years** | | | |  |
| --- | --- | --- | --- | --- | --- | --- | --- | --- | --- | --- |
| **Site** | **Medicine** | **Cases**  **n (%)** | **Controls**  **n (%)** | **Adjusted‡ OR (95%CI)** | **Adjusted‡ p-value** | **Cases**  **n (%)** | **Controls**  **n (%)** | **Adjusted‡ OR (95%CI)** | **Adjusted‡ p-value** | **Interaction test p-value** |
| **colon** | **any antibiotic use** | 35 (66·0%) | 82 (62·1%) | 1·98 (0·82,4·81) | 0·130 | 1,052 (63·7%) | 2,645 (63·1%) | 1·01 (0·87,1·16) | 0·920 | 0·139 |
|  | **anti-anaerobic activity *** | 27 (50·9%) | 67 (50·8%) | 1·27 (0·54,2·99) | 0·580 | 823 (49·8%) | 2,065 (49·3%) | 1·03 (0·90,1·18) | 0·690 | 0·629 |
|  | **non anti-anaerobic activity *** | 23 (43·4%) | 58 (43·9%) | 1·62 (0·69,3·76) | 0·265 | 697 (42·2%) | 1,807 (43·1%) | 0·87 (0·76,1·00) | 0·048 | 0·156 |
| **rectum** | **any antibiotic use** | 15 (50·0%) | 38 (57·6%) | 0·73 (0·16,3·38) | 0·689 | 439 (60·7%) | 1,076 (59·5%) | 1·12 (0·91,1·38) | 0·279 | 0·587 |
|  | **anti-anaerobic activity *** | 11 (36·7%) | 29 (43·9%) | 1·30 (0·37,4·63) | 0·682 | 342 (47·3%) | 851 (47·1%) | 1·02 (0·83,1·25) | 0·841 | 0·708 |
|  | **non anti-anaerobic activity *** | 9 (30·0%) | 23 (34·9%) | 0·51 (0·12,2·10) | 0·350 | 291 (40·3%) | 716 (39·6%) | 1·04 (0·84,1·28) | 0·712 | 0·328 |

Abbreviations: BMI: body mass index: OR: odds ratio, CI confidence interval; * primary clinical therapeutic effect on gut microbe; ‡ adjusted for diabetes, myocardial infarction, coronary heart disease, heart failure, peripheral vascular disease, dementia, cerebrovascular disease, chronic obstructive pulmonary disease, osteoporosis, renal disease, liver disease, hemiplegia/paraplegia, gallstones, acromegaly, low dose aspirin, NSAIDs and BMI.

**Table S9 Associations between antibiotic use and colorectal cancer, adjusting for comorbidities, medicine use, alcohol consumption, smoking and BMI**

|  |  | **<50 years** | | | | **>=50 years** | | | |  |
| --- | --- | --- | --- | --- | --- | --- | --- | --- | --- | --- |
| **Site** | **Medicine** | **Cases**  **n (%)** | **Controls**  **n (%)** | **Adjusted‡ OR (95%CI)** | **Adjusted‡ p-value** | **Cases**  **n (%)** | **Controls**  **n (%)** | **Adjusted‡ OR (95%CI)** | **Adjusted‡ p-value** | **Interaction test p-value** |
| **colon** | **any antibiotic use** | 32 (65·3%) | 72 (63·7%) | 2·63 (0·93,7·43) | 0·069 | 959 (65.0%) | 2,402 (65·6%) | 0·97 (0·83,1·14) | 0·735 | 0·064 |
|  | **anti-anaerobic activity *** | 24 (49.0%) | 59 (52·2%) | 1·55 (0·56,4·28) | 0·393 | 755 (51·2%) | 1,883 (51·4%) | 1·00 (0·86,1·16) | 0·990 | 0·399 |
|  | **non anti-anaerobic activity *** | 22 (44·9%) | 51 (45·1%) | 2·61 (0·95,7·15) | 0·062 | 638 (43·2%) | 1,655 (45·2%) | 0·85 (0·73,0·98) | 0·029 | 0·030 |
| **rectum** | **any antibiotic use** | 13 (50·0%) | 34 (64·2%) | 0.01 (0.00,3.10) | 0.182 | 386 (62·2%) | 967 (61·4%) | 1·08 (0·86,1·36) | 0·501 | 0·169 |
|  | **anti-anaerobic activity *** | 9 (34·6%) | 25 (47·2%) | 0·00 (0·00,2·60) | 0·089 | 302 (48·6%) | 765 (48·6%) | 1·00 (0·80,1·25) | 0·983 | 0·089 |
|  | **non anti-anaerobic activity *** | 8 (30·8%) | 20 (37·7%) | 0·54 (0·05,5·52) | 0·600 | 257 (41·4%) | 651 (41·7%) | 1·01 (0·80,1·27) | 0·937 | 0·691 |

Abbreviations: BMI: body mass index: OR: odds ratio, CI confidence interval; * primary clinical therapeutic effect on gut microbe; ‡ adjusted for diabetes, myocardial infarction, coronary heart disease, heart failure, peripheral vascular disease, dementia, cerebrovascular disease, chronic obstructive pulmonary disease, osteoporosis, renal disease, liver disease, hemiplegia/paraplegia, gallstones, acromegaly, low dose aspirin, NSAIDs, smoking status, alcohol use and BMI.
